# Role of Oxidative Stress on Vaginal Bleeding during The First Trimester of Pregnant Women

**Published:** 2013-12-22

**Authors:** Rüya Deveer, Mehmet Deveer, Yaprak Engin-Üstün, Eren Akbaba, Sema Uysal, Esma Sarikaya, Cavidan Gülerman, Leyla Mollamahmutoglu

**Affiliations:** 1 Department of Gynecology and Obstetrics, Dr. Zekai Tahir Burak Women’s Health Education and Research Hospital, Ankara, Turkey; 2 Department of Radiology, Sitki Kocman University Scool of Medicine, Mugla, Turkey; 3 Departmen of Biochemistry, Fatih University Scool of Medicine, Ankara, Turkey

**Keywords:** Oxidative Stress, Vaginal Bleeding, First Trimester

## Abstract

**Background:**

Reactive oxygen species (ROS) are produced in many metabolic and physiologic processes. Antioxidative mechanisms remove these harmful species. Our aim was to assess whether serum total antioxidant capacity and total oxidant status altered during first trimester pregnancies with vaginal bleeding.

**Materials and Methods:**

In this cross-sectional study, A group of pregnant women at less than 10 weeks of gestation with vaginal bleeding (n=25) and a control group of healthy pregnancies with similar characteristics (n=25) were included. All of the patients in the two groups were matched for age, gestational age and body mass index. Serum total antioxidant capacity and total oxidant status levels were determined using a Hitachi 912 analyzer and compared between the two groups.

**Results:**

Characteristics, including maternal age, parity, and gestational age were similar between the two groups. Serum total antioxidant capacity levels were significantly lower in the women with vaginal bleeding than in control women (1.16 ± 0.20 vs. 1.77 ± 0.08 mmol Trolox Equiv./L; p=0.001), whereas higher total oxidant status measurements were found in women with vaginal bleeding compared to the control group (4.01 ± 0.20 vs. 2.57 ± 0.65 µmol H_2_O_2_ Equiv./L; p=0.001).

**Conclusion:**

Increased total oxidant status might be involved in the pathophysiology of vaginal bleeding during early first trimester pregnancies.

## Introduction

Oxygen undergoes extensive metabolism that can result in the production of toxic derivatives. Activated molecular species derived from oxygen metabolism are designated as reactive oxygen species (ROS, 1). The ROS molecules are generated within the mitochondrial respiratory system cells as by-products of aerobic respiration and metabolism. Excess of ROS production leads to cell damage and cell dysfunction; cells have evolved antioxidant systems to prevent ROS-induced damage (2, 3). Oxidative stress of placenta plays an important role in the pathogenesis of many pregnancy complications including miscarriages, preeclampsia and preterm labor (4, 5). Disturbed oxidantantioxidant system balance may induce damage to developing pregnancy in which the bleeding may be the first sign. Our aim was to assess the status of serum total antioxidant capacity (TAC) and total oxidant status (TOS) in pregnant women presenting with light vaginal bleeding during the first trimester of pregnancy.

## Materials and Methods

A cross-sectional study was carried out in 50 women who received antenatal and obstetric care at Perinatology Unit of Zekai Tahir Burak Women Health Education and Research Hospital between January and May 2011. This study was approved by The Medical Ethics Committee of the hospital, and informed consent was obtained from all of the participants. Subjects were eligible for enrollment if they were between 16 and 45 years of age. Gestational age was evaluated on the basis of the last menstrual period and confirmed by ultrasound. Patients were divided into two groups.

### Study group

The first group included 25 pregnant women at less than 10 weeks’ gestation with light, intermittent, painless vaginal bleeding. This was determined by the clinical history and clinical examination which included gynecologic examination and trans-vaginal ultrasonography. All these ended in an uneventful pregnancy at term, with a normal baby. Complete bed-rest at home was recommended in all cases with vaginal bleeding. All patients were followed at 7 day intervals clinically, including bimanual examination and sonographically, until the bleeding stopped.

### Control group

The second group consisted of 25 patients at less than 10 weeks of gestation with normal ongoing pregnancies. The pregnant controls were selected from the ones at less than 10 gestational weeks with no pre-existing complications. A gestational sac with fetal heart rate was identified by trans-vaginal ultrasonography. All of the patients in the control group were matched for age, gestational age and body mass index (BMI).

Exclusion criteria were as follows: gestational age after 10 weeks (based on the 1^st^ day of the last menstrual period; n=3), history of recurrent spontaneous miscarriages (defined as three or more consecutive pregnancy losses; n=2), history of documented chromosomal abnormalities, endocrine diseases (n=2), internal diseases, connective tissue diseases, hypertension, coagulopathies, multiple pregnancies (n=1), smoking (n=1), diabetes mellitus, and anemia (n=2). A total of 61 patients were screened, but 50 (81.96%) of them met our selection criteria.

All blood samples were obtained before administration of any medication and before any medical or surgical intervention. Serum was separated by centrifugation at 4000 g for 10 minutes and frozen at -70˚C for later analysis. Serum TAC and TOS levels were assayed using a Hitachi 912 analyzer (Roche Diagnostics, Geneva, Switzerland).

### Measurement of total antioxidant capacity

Serum TAC was determined using an automated measurement method, developed by Erel (6). In this method, hydroxyl radical is produced. Ferrous ion solution, which is present in reagent 1, is mixed with hydrogen peroxide, which is present in reagent 2. The sequential process produced radicals such as brown-colored dianisidinyl radical cation, are potent radicals. This method measures the antioxidative effect of the sample against the potent free radical reactions, which is initiated by the produced hydroxyl radical. The results are expressed as mmol trolox equiv./l.

### Measurement of total oxidant status

Serum TOS was determined using a novel automated measurement method, developed by Erel (7). Oxidants oxidize the ferrous ion-o-dianisidine complex to ferric ion. Glycerol molecules enhance the oxidation reaction. In an acidic medium, the ferric ion makes a colored complex with xylenol orange. The color intensity can be measured spectrophotometrically and it is related to the total amount of oxidant molecules present in the sample. Hydrogen peroxide is used for the calibration, and the results are expressed in terms of micromolar hydrogen peroxide equivalent per liter (μmol H<sub>2</sub>O<sub>2</sub> equiv./l).

The SPSS package for windows version 15.0 software (SPSS Inc, Chicago, IL, United States) was used to perform statistical analyses. Distribution of the groups was analyzed with one sample Kolmogrov-Smirnov test. All data were distributed normally. Students’ two-tailed-t test was used for the assessment of differences between groups. A probability p value of <0.05 was considered statistically significant.

## Results

Details of pregnancies including gestational age and BMI are shown in table 1. There were no statistically significant differences between two groups regarding maternal age, number of pregnancies, gestational age and BMI (Table 1).

Maternal serum TAC levels were significantly lower in pregnancies with vaginal bleeding compared to controls (p=0.001), whereas TOS values were significantly higher in pregnancies with vaginal bleeding than controls (p=0.001, Table 1).

**Table 1:** Baseline characteristics and output data of the women


Characteristics	Study group (n=25)	Control group (n=25)	P value

Maternal age (Y)*	29.48 ± 5.46	28.56 ± 5.47	0.555
BMI (kg/m²)*	24.76 ± 4.02	23.11 ± 4.41	0.173
Gravida**	2 (1-6)	2 (1-5)	0.308
Gestational age (weeks)*	8.13 ± 1.87	8.46 ± 2.93	0.635
**TAC (mmol Trolox Equiv. /L)**	1.16 ± 0.20	1.77 ± 0.08	0.001
**TOS (µmol H<sub>2</sub>O<sub>2</sub>Equiv./L)**	4.01 ± 0.20	2.57 ± 0.65	0.001


*; Values are mean ± SD, **; Values are median (range) SD; Standard deviation, BMI; Body mass index, TAC; Total antioxidant capacity and TOS; Total oxidant status.

## Discussion

The current study showed that serum TOS levels were higher and serum TAC levels were lower in pregnant women with vaginal bleeding when compared to women with normal-ongoing pregnancies of similar gestational age in the first trimester. Vaginal bleeding is common complication in the first trimester of pregnancies and may be an early marker for placental dysfunction. About half of patients presenting with bleeding have miscarriage (8). Still, the precise etiology of spontaneous abortion remains unclear. About 50% of all spontaneous abortions are associated with chromosomal abnormalities. The remaining 50% of the causes may be preventable (9). Pregnancies with vaginal bleeding have increased risk of other adverse outcomes such as placental abruption, low birth weight and preterm delivery (10-12). There is now compelling evidence that oxidative stress is one of the main underlying mechanisms in the pathogenesis of spontaneous abortion (13). Oxidative stress is well known to initiate the caspase cascade leading to cell death in other systems. Concentrations of lipid peroxides increase in the decidua of women undergoing early pregnancy loss (14). Current evidence reveals that the architecture of the human first-trimester gestational sac limits fetal exposure to oxygen and tries to minimize the damage caused by oxygen free radicals. Failure of placentation is associated with an imbalance in ROS, which will further affect placental development and function and may subsequently have an influence on both the fetus and its mother (15, 16). During the embryonic period of pregnancy the prevailing oxygen tension is low and metabolism is mainly anaerobic (17). Thus the production of ROS is reduced. Oxidative stress in early pregnancy may lead to complications such as recurrent abortions, preeclampsia and congenital anomalies in diabetes (18). Teratogenic drugs can also induce embryotoxicity through ROS-mediated oxidative stress (19).

Antioxidants can exist in enzymatic and nonenzymatic forms. Common enzymatic defenses include superoxide dismutase (SOD), catalase, and glutathione peroxidase and glutathione reductase. Nonenzymatic agents are ferritin, ceruloplasmin, transferin, ascorbic acid (vitamin C), and [alpha]- tocopherol (vitamin E).

A previous study by Ozkaya et al. (20) showed that early spontaneous abortions accompanied by vaginal bleeding were associated with increased serum malondialdehyde levels and decreased SOD levels.

In the present study decreased serum total antioxidant capacity levels and increased serum total oxidant status in the pregnant women at less than 10 weeks of gestation with light vaginal bleeding compared with the normal pregnant women were determined using a novel automated measurement method (6, 7). It is currently unclear where the oxidative stress occurs. It is not known exactly whether oxidant/antioxidant imbalance is the consequence or the cause in the development of vaginal bleeding. All the patients in the study ended in an uneventful pregnancy at term, with a normal baby. None of them had any adverse pregnancy outcome.

There are several limitations of our study. The sample size of the study is relatively small and the design is cross-sectional in nature. Moreover it is difficult to justify the role of antioxidant supplementation in the prevention of ROS- induced damage, as none of our subjects received vitamin supplementation before or in the early weeks of their pregnancies. A third group of women with vaginal bleeding ending with abortion can be evaluated and this examination could help to determine whether there is a cut off level for TOS and TAC in early pregnancy which discriminates between viable and non-viable pregnancy.

## Conclusion

The possible subsequent outcomes associated with oxidant/antioxidant imbalance in early pregnancies with vaginal bleeding remain to be established. Further well-designed randomized control studies are needed to determine a threshold value for TOS and TAC levels. Also the effectiveness of antioxidant supplementation in reversing spontaneous abortions needs to be established.
